# Supplementary material for: Predicting vaccine effectiveness for mpox
Source: Nat Commun. 2024 May 8;15:3856. doi: 10.1038/s41467-024-48180-w (PMC11078999; doi:10.1038/s41467-024-48180-w)
Supplement: Supplementary file 1 — Supplementary Information [file 41467_2024_48180_MOESM1_ESM.pdf]

Supplementary Information

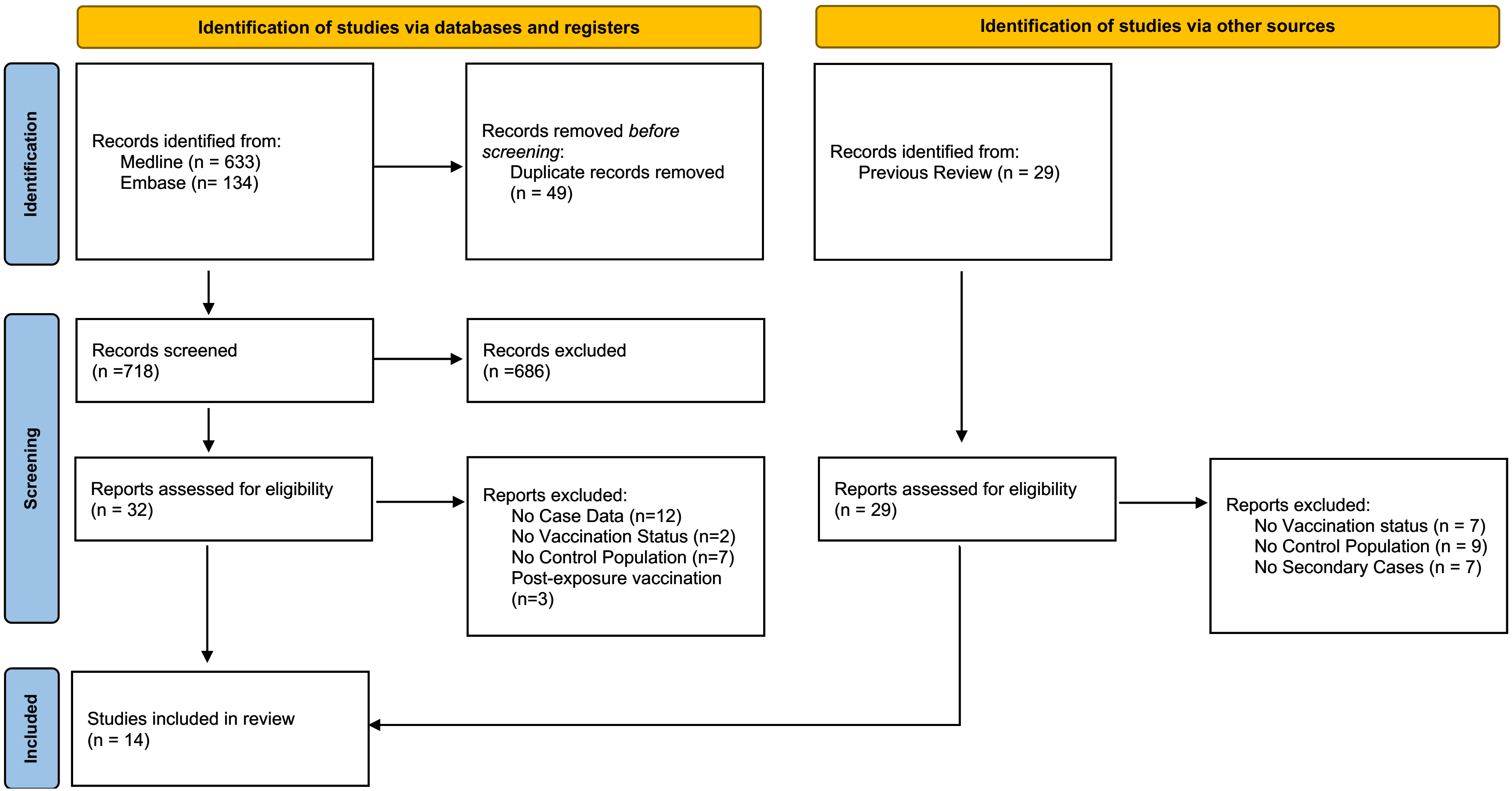

Figure S1: PRISMA flowchart for studies identified through a systematic review process into the effectiveness of different Mpox vaccines. 8 studies were included from our systematic search and 6 from a previous review.

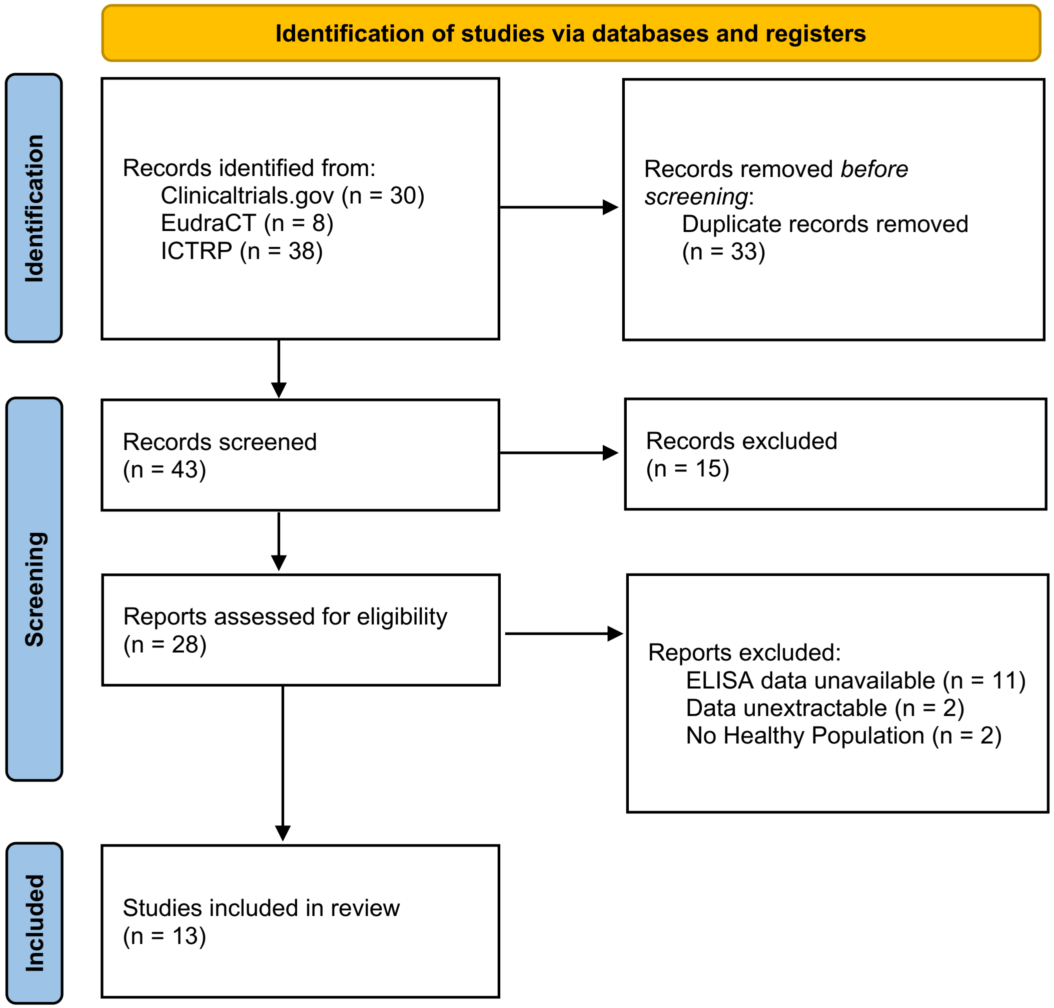

Figure S2: PRISMA flowchart for the immunogenicity studies identified as part of the systematic review process.

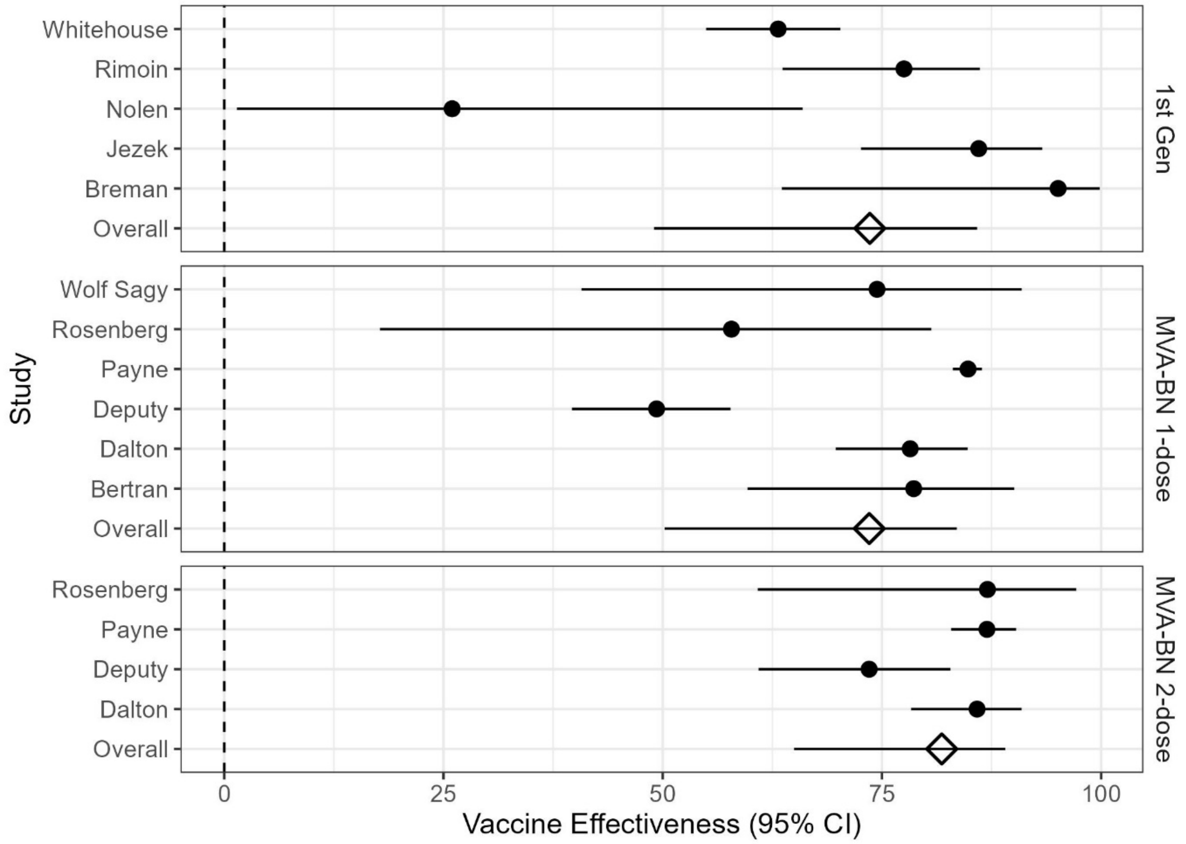

Figure S3: Forest plot comparing the estimated vaccine effectiveness within each of the different studies to assess the heterogeneity. Overall estimates for each vaccine are indicated by a diamond, the estimated effectiveness within each study is indicated by the circle (median of the posterior distribution) with horizontal bars representing the 95% credible interval.

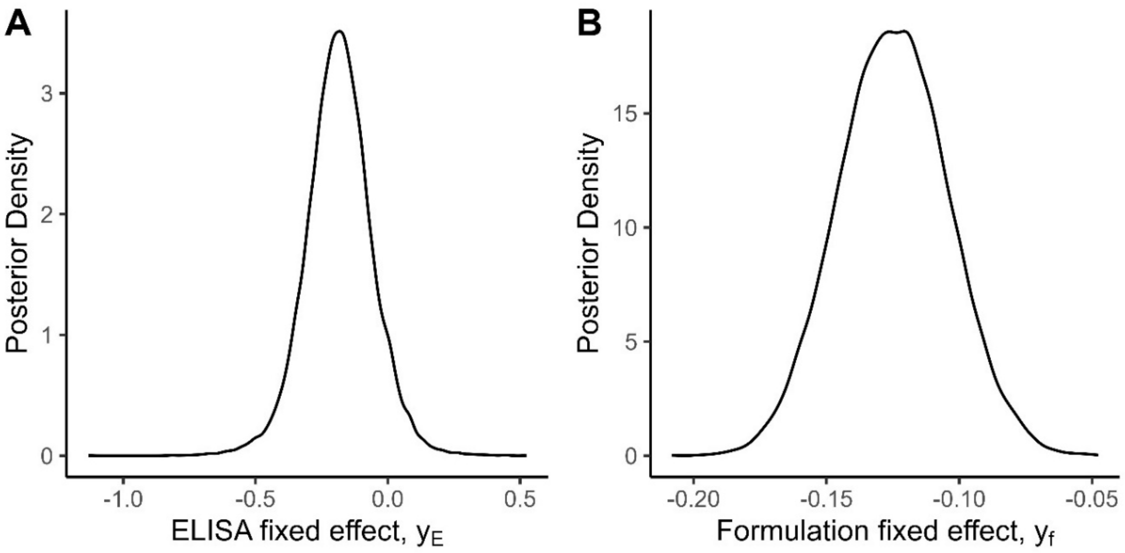

Figure S4: The posterior distributions of the fixed effects on the (log_{10}) GMT in the immunogenicity model (n=12 Trials). (A) The effect of the ELISA assay was not found to be significant (Fold Difference (10^{μ_E}) 1.3- fold, CI:0.54-3.48). (B) The effect due to different formulations of MVA-BN was found to be significant (Fold Difference (10^{μ_f}) 1.33-fold CI:1.21-1.46).

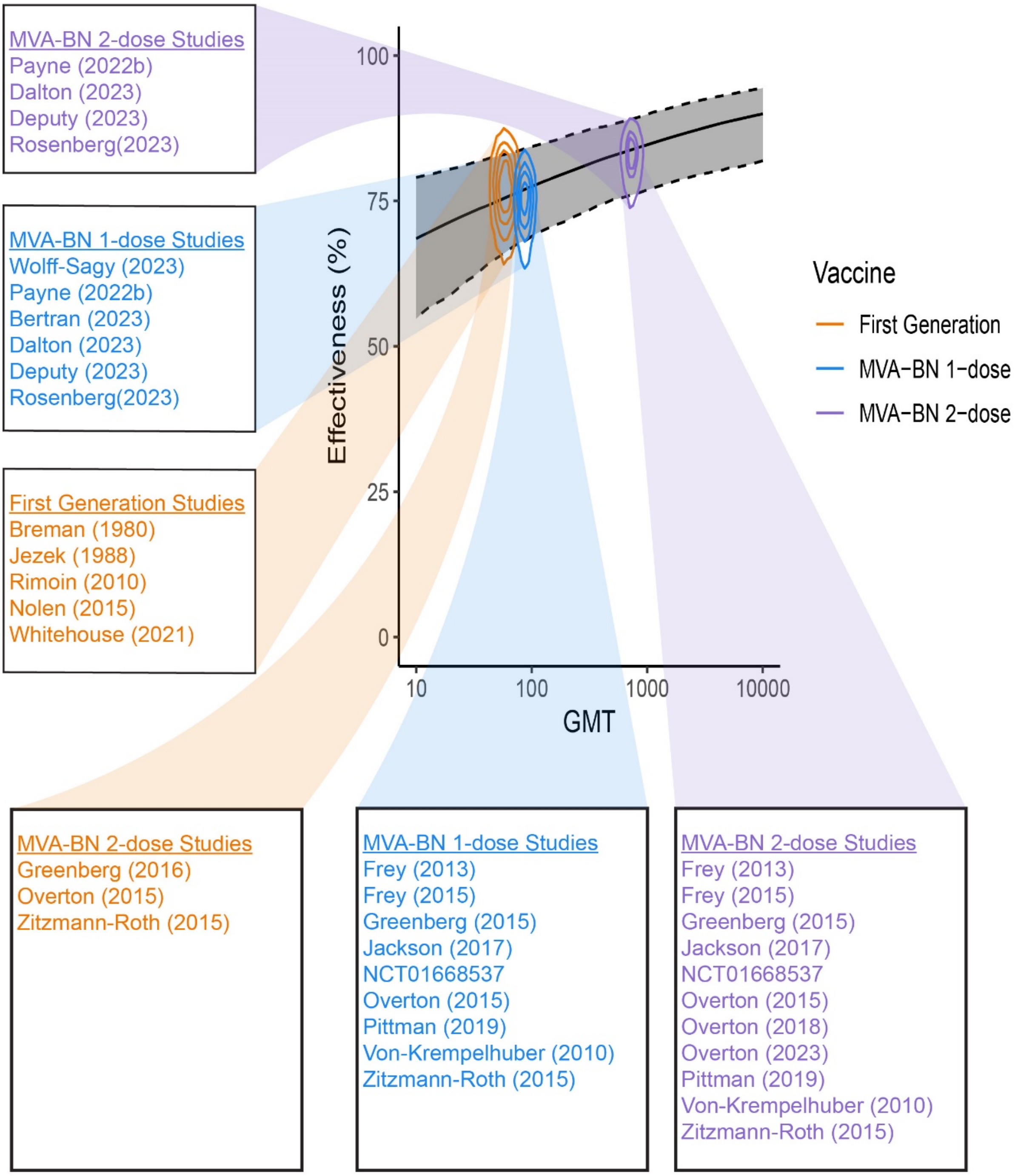

Figure S5: Reproduced figure of the logistic model relating the effectiveness data (n=11) and the immunogenicity data (n=12) (i.e. Figure 3), with annotation of the studies that contribute to each of the data points.

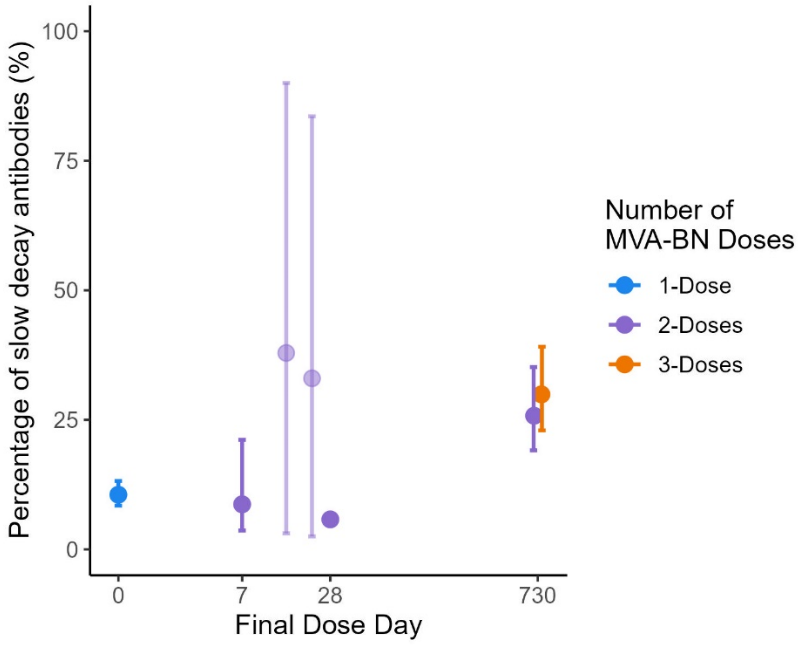

Figure S6: The estimated proportion of slow-decaying (long-lived) antibodies (circles, median of the posterior distributions) and 95% credible intervals (error bars) induced from vaccination with different dosing schedules using the same model as in Figure 4a (n=13 Trials). Primary doses are administered on day 0, with the three-dose schedule including a second dose 28-days after the primary dose. Percentages for regimens without a datapoint later than 5 months post-vaccination are shown with reduced opacity. 2-Doses with the final dose on day 28 has a narrow credible interval, which is obscured in the figure.

Effectiveness Study	Vaccine	Country	Study Design	Observation period	Follow-up *	Population/ Control	Total cases	Disaggregation[#]	Reported Doses	Included in Data Analysis^
Breman 1980[1]	First Generation	DRC + Nigeria	Secondary Contacts	1970-1979	Not reported	447	4	Spatial	At least 1	Included
Fine 1988[2]	First Generation	DRC	Secondary Contacts	1980-84	Not reported	834	36	Spatial	At least 1	Not included. Major overlap with (Jezek 1988)
Jezek 1986[3]	First Generation	DRC	Secondary Contacts	1980-1984	Not reported	2510	56	Spatial	At least 1	Not included. Major overlap with (Jezek 1988)
Jezek 1988[4]	First Generation	DRC	Secondary Contacts	1981-1986	Not reported	2278	69	Spatial and age	At least 1	Included
Rimoin 2010[5]	First Generation	DRC	Case-Coverage	2005-2007	Not reported	Estimated	760	Age	At least 1	Included
Nolen 2015[6]	First Generation	DRC	Secondary contacts	2013 (Jul-Dec)	Not reported	97	44	None	At least 1	Included
Whitehouse 2021[7]	First Generation	DRC	Case-Coverage	2011-2015	Not reported	Estimated	1057	None	At least 1	Included
Wolff Sagy 2023[8]	MVA-BN	Israel	Cohort Study	2022 (Aug-Nov)	90-147 days	2054	18	Weekly	At least 1	Included
Payne 2022a[9]	MVA-BN	US	Case-Coverage	2022 (Jul-Sep)	Not reported	Estimated	5402	Weekly	At least 1	Not included. Major overlap

										with (Payne 2022b)
Payne 2022b[10]	MVA-BN	US	Case-Coverage	2022 (Jul-Oct)	Not reported	Estimated	9544	Weekly	1 or 2	Included
Bertran 2023[11]	MVA-BN	UK	Case-Coverage	2022 (Jul-Oct)	Not reported	89240	460	Weekly	At least 1	Included
Dalton** 2023[12]	MVA-BN	US	Case-Control	2022-2023 (Aug-Mar)	Not reported	608	309	None	1 or 2	Included
Deputy** 2023[13]	MVA-BN	US	Case-Control	2022 (Aug-Nov)	Not reported	8649	2266	None	1 or 2	Included
Rosenberg** 2023[14]	MVA-BN	US	Case-Control	2022 (Jul-Oct)	Not reported	507	252	None	1 or 2	Included

Table S1: Summary of vaccine effectiveness studies identified from the systematic search and that were considered for analysis.

* Follow-up time refers to the time interval in which cases were recorded after vaccination.

# Spatial disaggregation split secondary contacts between household and non-household contacts, and in some cases by proximity of household to primary case (same house, neighbouring house, other house in village or other village).

^ The indicated studies were excluded from analysis since they were highly overlapping with other studies included in the analysis and since a more complete data was available from another related study.

** Dalton, Deputy and Rosenberg all use public databases of Mpox cases from similar regions and likely contain overlapping case data but due to significant differences in methodology for determining the control group, the studies were all included in the analysis.

Clinical Trial Identifier	Participants	Prior Vaccinia Status	Medical Conditions	Doses	Age	1 Dose MVA-BN	2 Dose MVA-BN	Weeks sampled after first dose	ELISA Assay OD#	Formulation^	Published article
NCT00189956	165	Naïve	Healthy	2	18-30	Yes	Yes	4,6,12	0.35	FD	von Krempelhuber 2010[15]
NCT00316524	745	Experienced and Naive	Healthy	0-2	18-55	Yes	Yes	4,6,8,30,106	NR	LF	Zitzmann-Roth 2015[16]
NCT01913353	440	Naïve	Healthy	2	18-42	Yes	Yes	4,6,8	NR	LF	Pittman 2019[17]
NCT00316602	632	Naïve	AD and healthy	2	18-40	Yes	Yes	4,6,8,32	0.3	LF	Greenberg 2015[18]
NCT00437021	206	Naïve	Healthy	1,2	18-38	Yes	Yes	3,4,5,6,8,26,52	0.35	LF	Frey 2013[19]
NCT00316589	581	Experienced and Naive	HIV and healthy	2	18-55	Yes	Yes	4,6,8,32	0.3	LF	Overton 2015[20]
NCT01144637	4005	Naïve	Healthy	2	18-40	No	Yes	6	NR	LF	Overton 2018[21]
NCT00686582	304	Naïve	Healthy	3	18-35	Yes	Yes	106,108,134	0.35	LF	Ilchmann 2023[22]
NCT00857493	120	Experienced	Healthy	2	56-80	Yes	Yes	2,4,6,8,28,32	0.3	LF	Greenberg 2016[23]
NCT01668537	651	Naïve	Healthy	2	18-55	Yes	Yes	4,6,8	NR	LF and FD	Published online only[24]
NCT00914732	523	Naïve	Healthy	2	18-38	Yes	Yes	4,6,8,30	0.35	LF and FD	Frey 2015[25]
NCT03699124	1129	Naïve	Healthy	2	18-45	No	Yes	6	NR	FD	Overton 2023[26]
NCT01827371	435	Naïve	Healthy	2	18-40	Yes	Yes	4,5,6,7,8	0.3	FD	Jackson 2017[27]

Table S2: Summary of vaccine immunogenicity trials included in our meta-analysis. Data was extracted from the clinical trials registry or, when not present in the registry, by identifying a linked research article where trial results were disseminated.

#ELISA assays were conducted at either: (a) wavelength 450nm/endpoint optical density (OD) = 0.3, or (b) wavelength 492nm/endpoint OD= 0.35. Trials where the cut-off was not reported are listed as NR.

^FD=Freeze Dried, LF=Liquid Frozen.

Parameter		Symbol	Estimate (95% Credible Interval)	Prior
Effectiveness	First Generation	E_1	0.74 (0.49-0.86)	$log(1-E_1) \sim U(0,1)$
	1-Dose MVA-BN	E_2	0.74 (0.50-0.84)	$log(1-E_2) \sim U(0,1)$
	2-Dose MVA-BN	E_3	0.82 (0.65-0.89)	$log(1-E_3) \sim U(0,1)$
Interstudy Standard deviation		σ_E	0.5 (0.28-1.11)	Half-Cauchy(0,0.25)

Table S3: Summary of the estimated effectiveness parameters and the prior distributions. Risk of infection $r_{i,v,s}$, and the random study effects, S_s, were also estimated and can be accessed using the GitHub repository.

Parameter		Symbol	Estimate (95% Credible Interval)	Prior
$\log_{10}$(GMT)	First Gen	μ_1	1.77 (1.61-1.91)	Normal(0,10)
	1-Dose MVA-BN	μ_2	1.94 (1.82-2.06)	Normal(0,10)
	2-Dose MVA-BN	μ_3	2.87 (2.75-2.98)	Normal(0,10)
Standard deviation of ($\log_{10}$) titers	First Gen	σ_1	0.765 (0.710-0.827)	Log-normal(0,10)
	1-Dose MVA-BN	σ_2	0.662 (0.643-0.683)	Log-normal(0,10)
	2-Dose MVA-BN	σ_3	0.386 (0.378-0.394)	Log-normal(0,10)
Formulation Effect		μ_f	0.13 (0.08-0.17)	Normal(0,1)
ELISA effect		μ_E	0.14 (-0.27-0.54)	Normal(0,1)
Interstudy Standard Deviation		σ_I	0.17 (0.11-0.30)	Half-Cauchy(0,1)

Table S4: Summary of the parameters used to fit the immunogenicity data. The estimates of the individual study effects are not included in the table.

Parameter		Symbol	Estimate (95% Credible Interval)
$\log_{10}$(GMT)	First Gen	μ_1	1.77 (1.61-1.91)
	1-Dose MVA-BN	μ_2	1.94 (1.83-2.05)
	2-Dose MVA-BN	μ_3	2.87 (2.76-2.98)
Standard deviation of ($\log_{10}$) titers	First Gen	σ_1	0.77 (0.72-0.84)
	1-Dose MVA-BN	σ_2	0.67 (0.65-0.69)
	2-Dose MVA-BN	σ_3	0.39 (0.38-0.40)
Formulation Effect		μ_f	0.13 (0.08-0.17)
Logistic Slope		k	0.49 (0.21-0.79)
Logistic Constant		A	1.44 (0.89-1.92)
Interstudy Standard Deviation	Immunogenicity	σ_I	0.17 (0.11-0.30)
	Efficacy	σ_E	0.47 (0.26-0.93)

Table S5: The estimated parameters from the fitted logistic model relating data on vaccine immunogenicity and effectiveness in Figure 3.

Parameter	Dosing Scheme	Symbol	Prior	Estimate (95% Credible Interval)
Initial GMT	1-Dose	x_0	$\log(x_0) \sim N(0,10)$	80 (63-102)
	2-Dose (28 days)			673 (537-854)
	2-Dose (730 days)			2136 (1596 -2864)
	3-Dose			2211 (1685
Slow-decay rate (long-lived antibodies)		δ_l	$\delta_l \sim N(0,1)$	0.0028 (0.0007- 0.0050)
Fast-decay rate (short-lived antibodies)		δ_s	$\delta_l \sim N(0,1)$	0.23 (0.20-0.27)
Proportion of fast decaying antibodies	1-Dose	f	$f \sim U(0,1)$	0.89 (0.87-0.92)
	2-Dose (28 days)			0.94 (0.93-0.95)
	2-Dose (730 days)			0.74 (0.65-0.81)
	3-Dose			0.70 (0.61-0.77)

Table S6: The estimated decay parameters from fitting a dual exponential model to vaccine immunogenicity data.

Model	LPPD	P_{waic}	WAIC
Two Phase Decay Model with distinct decay rates	-1756.6	199.6	3912
Two Phase Decay Model with common decay rates	-1756.9	196.8	3907
Single Phase Decay Model with distinct decay rates	-2435.4	744.0	6359
Single Phase Decay Model with common decay rate	-2676.1	683.3	6718
No Decay Model	-3422.1	839.1	8522

Table S7: Comparison of the Widely Applicable Information Criterion (WAIC) for different models of the decay in antibody binding. Higher log-posterior predictive density (LPPD) indicates a greater predictive fit. The P_{waic} is a penalty term.

Vaccination Schedule	Predicted ELISA GMT (95% Credible Interval) at time points:				
	3 months	6 months	12 months	18 months	24 months
1-Dose	12 (9-15)	8 (6-11)	7 (5-10)	7 (5-9)	6 (5-9)
2-Dose (Day-28)	68 (52-91)	38 (30-48)	34 (26-43)	31 (24-41)	29 (22-39)
2-Dose (Day-730)	610 (451-834)	517 (375-720)	476 (342-670)	441 (314-633)	410 (286-601)
3-Dose	714 (535-959)	619 (458-844)	571 (419-785)	530 (384-742)	493 (347-705)

Table S8: The predicted vaccinia-binding GMT at different time points after the peak titer using the fitted model in Figure 4a. We compare the effect of different vaccination schedules on the expected titer.

Parameter		Symbol	Prior
$\log_{10}$(GMT)	First Gen	μ_1	Normal(0,10)
	1-Dose MVA-BN	μ_2	Normal(0,10)
	2-Dose MVA-BN	μ_3	Normal(0,10)
Standard deviation of ($\log_{10}$) titers	First Gen	σ_1	Log-normal(0,10)
	1-Dose MVA-BN	σ_2	Log-normal(0,10)
	2-Dose MVA-BN	σ_3	Log-normal(0,10)
Formulation Effect		μ_f	Normal(0,1)
Logistic Slope		k	Normal(0,1)

Logistic Constant		A	$\mathrm{Logistic}(0,1)$
Interstudy Standard Deviation	Immunogenicity	σ_I	$\text{Half-Cauchy}(0,1)$
	Efficacy	σ_E	$\text{Half-Cauchy}(0,0.25)$
Risk of Vaccination/Infection		$r_{i,v,s}$	$\mathrm{Beta}(1,1)$
Study Effects*	Immunogenicity	μ_I	$\mathrm{Normal}(0, \sigma_I)$
	Efficacy	μ_E	$\mathrm{Normal}(0, \sigma_E)$

Table S9: List of all the model parameters and priors used to fit a logistic relationship between efficacy and immunogenicity data. The study effects are model parameters within a hierarchical structure and the reported prior is the modelled hierarchy. *Study Effects are latent variables which are estimated but fall under the hierarchical model and the distribution in the prior column is the hierarchical structure rather than priors.

Supplementary Methods:

Details of Systematic Search

Effectiveness Search

Our systematic search for vaccine effectiveness studies extended the search from Bunge et al.[28] by adding the search terms “AND (Vaccination[tiab] OR Vaccine[tiab] OR Immunisation[tiab] OR Immunization[tiab])” and “OR Mpox[tiab]”. These additional terms limited the scope to papers that considered vaccination usage and updated the search for the change in name. We conduct this search from the date, 7th September 2020 to the 10th July 2023. Specifically, we searched the PubMed (including the MEDLINE database) and Embase (Ovid) databases, with the search terms:

PubMed:

(Monkeypox[MeSH] OR “Monkeypox virus”[MeSH] OR monkeypox[tiab] OR “monkey pox”[tiab] OR“variole du singe”[tiab] OR “variole simienne”[tiab] OR Mpox[tiab])AND (Vaccination[tiab] OR Vaccine[tiab] OR immunisation[tiab] OR immunization[tiab]) ;

Embase (Ovid):

(’monkeypox’/exp OR ’monkeypox virus’/exp OR monkeypox:ti,ab OR "monkey pox":ti,ab OR mpox:ti,ab) AND (Vaccinatio:ti,ab’OR Vaccine:ti,ab OR Immuni#ation:ti,ab)

In order to calculate vaccine effectiveness from a given study, and thus include the study in our analysis, we required the following data:

- In the case of studies based on population surveillance data (case-coverage), the incidence of infection must be provided in both vaccinated and unvaccinated at-risk groups.
- In the case of secondary-contact studies, the total number of secondary-contacts/exposed individuals (disaggregated by vaccine status) must be provided, along with the number of secondary-cases in vaccinated and unvaccinated individuals.

    - In the case of cohort studies, the cohort size of both vaccinated and unvaccinated groups must be provided along with the number of infections in each group.
    - For case-control studies we require a break-down of infection data by vaccination status along with an uninfected control group split by vaccination status as a comparator.

Immunogenicity Search

To obtain immunogenicity data we conducted a systematic search of registered clinical trials from the clinicaltrials.gov, EudraCT and ICTRP databases with the search terms;

clinicaltrials.gov:

- Intervention: MVA
- Condition: Smallpox OR Monkeypox OR Variola;
- Study Type: Interventional

EudraCT and ICTRP;

MVA AND (Smallpox Or Monkeypox OR Variola).

Studies were included if they met the following criteria:

- Vaccination was with the MVA-BN vaccine
- At least one arm contained a healthy population.
- Vaccinia-specific binding antibody titers were measured via an ELISA

Derivation of Binomial model for test-negative case-control studies:

Let $P(V|I) = \phi$ be the probability that a person is vaccinated (V) given they are infected (I). Then,

$$P(I|V) = \frac{P(V|I)P(I)}{P(V)}. \qquad S1$$

The calculation for $P(I|U)$ (Probability of infection given unvaccinated (U)), is similar and we can then calculate the ratio,

$$\frac{P(I|V)}{P(I|U)} = \frac{P(V|I)}{P(U|I)}\frac{P(U)}{P(V)}. \qquad S2$$

Conducting a similar calculation for the control group, let $P(V|C) = \gamma$, where C is the control group, then,

$$1 - E = \frac{P(I|V)}{P(I|U)} = \frac{\phi}{1-\phi}\frac{1-\gamma}{\gamma}\frac{P(C|V)}{P(C|U)}. \qquad \text{S3}$$

In an ideal test-negative design study the fraction $\frac{P(C|V)}{P(C|U)} = 1$, however, unmeasured confounding can cause this quantity to vary around 1. Since this cannot be quantified directly, we incorporate this ratio into a random effect with $S = \log\frac{P(C|U)}{P(C|V)}$. Taking logarithms of Equation S3 we obtain,

$$\mathrm{logit}(\phi) = \mathrm{logit}(\gamma) + \log(1 - E) + S. \qquad S4$$

This is equivalent to a model where the relative risk reduction of the vaccine has a random effect between studies. Comparing this model to the model used for case-coverage studies (equation 2 of the Methods) we find that both study types are described by applying the same model, except here $r_{v,0,s} = \gamma$, and $r_{v,i,s} = \phi$, whereas in the case-coverage method (from equation 2 of Methods), $r_{i,0,s} = \gamma$, and $r_{i,v,s} = \phi$. That is, for test-negative studies,

$$\mathrm{logit}(r_{v,i,s}) = \mathrm{logit}(r_{v,0,s}) + \log(1 - E_v) + S_s, \qquad S5$$

where $r_{v,0,s}$ and $r_{v,i,s}$ denote the probability of being vaccinated in the control and infected cohorts, respectively. The indexation in this case differs slightly from the case-coverage methods. That is, here the different disaggregation groups correspond to the vaccine (v), while the control and infectious groups are indexed by (i).

Immunogenicity data:

The immunogenicity data reported included geometric mean titers and confidence intervals. Using the confidence intervals reported, we calculate the approximate sample standard deviation, s, from each study and group assuming confidence intervals were calculated using the formula,

$$\mathrm{CI} = \log(GMT) \pm t_{0.05,n}\frac{s}{\sqrt{n}}, \qquad S6$$

where n is the sample size, GMT is the reported geometric mean titer, $t_{0.025,n}$ is the 0.025 percentile of the t-distribution with n-degrees of freedom. Due to rounding of the reported values, using the upper or lower confidence limits yielded different results. Hence, we

average across calculating the sample standard deviation estimates calculated from the upper and lower confidence limits.

In our evaluation of the posterior distribution, we used the sample mean (log) antibody titer, $\bar{y}_{s,v,f,E}$, and sample standard deviation of these (log titers), $\overline{sd}_{s,v,f,E}$ (for each vaccine, v, from each study, s, with vaccine formulation, f, and ELISA assay, E), rather than the individual titers (individual titers were not provided upon request). These are sufficient statistics (as will be seen below) to define our distribution of interest.

For simplicity, let $\mu_{s,v,f,E}$ denote the mean a cohort from the same study using the same vaccine (also using the same ELISA and formulation). That is, $\mu_{s,v,f,E} = \mu_v + \mu_E + \mu_f + \mu_s$, where, μ_v, is the mean of the 'true' distribution of titers (administered with liquid frozen formulation), μ_E, is the effect of the ELISA assay on the observed titers, μ_f, is the effect of using a freeze dried formulation of the vaccine and μ_S, is the random study effect on the observed titers. Then the likelihood of observing a particular set of individuals (log) antibody titers, $\boldsymbol{y}_{s,v,f,E}$, from a particular study/group/etc, given a certain (as yet to be estimated) $\mu_{s,v,f,E}$ and σ_v (standard deviation of the titers from the vaccine group) is given by,

$$p(\boldsymbol{y}_{s,v,f,E} | \mu_{s,v,f,E}, \sigma_{\mathrm{v}}) \propto \prod_i \frac{1}{\sigma_v \sqrt{2\pi}} \exp\left(\frac{-1}{2}\left(\frac{\mathrm{y_i} - \mu_{s,v,f,E}}{\sigma_v}\right)^2\right) \qquad S7$$

where y_i are the individual titers of $\boldsymbol{y}_{s,v,f,E}$. Rearranging this equation, we can write this as,

$$p(\boldsymbol{y}_{s,v,f,E} | \mu_{s,v,f,E}, \sigma_v) \propto \frac{1}{\sigma_v^n} \exp\left(\frac{-1}{2\sigma_v^2} \sum_i (y_i - \mu_{s,v,f,E})^2\right). \qquad S8$$

Constant terms can be ignored as we only require a proportional function to sample from the posterior[29]. Focusing on the summation in the exponent and using the definition of a sample standard deviation and sample mean, we can expand the summation and simplify obtaining,

$$\sum_i (y_i - \mu_{s,v,f,E})^2 = (n_{s,v,f,E} - 1)\overline{sd}^2 + n_{s,v,f,E}(\bar{y}_{s,v,f,E} - \mu_{s,v,f,E})^2, \qquad S9$$

where $n_{s,v,f,E}$ is the number of individual titers used in the sample mean and standard deviation for the given vaccine, study, formulation and ELISA combination. It follows that we

only need the sample mean, $\bar{y}_{s,v,f,E}$, and sample standard deviation, $\overline{sd}_{s,v,f,E}$, as sufficient statistics, to evaluate the likelihood function. The contributions of each cohort are multiplied together, yielding the full likelihood,

$$p\left(\bar{y}_{s,v,f,E}, \overline{sd}_{s,v,f,E} \middle| \mu_{s,v,f,E}, \sigma_v, n_{s,v,f,E}\right) \propto \prod \frac{1}{\sigma_v^{n_{s,v,f,E}}} \exp\left(\frac{-1}{2\sigma_v}\left((n_{s,v,f,E} - 1)\overline{sd}_{s,v,f,E} + n_{s,v,f,E}\left(\bar{y}_{s,v,f,E} - \mu_{s,v,f,E}\right)\right)\right)$$

The posterior distribution for all the parameters can then be determined using this likelihood function in conjunction with the priors (specified in the main text Methods).

References:

1. Breman, J. G. *et al.* Human monkeypox, 1970-79. *Bull World Health Organ* **58**, 165-182 (1980).
2. Fine, P. E., Jezek, Z., Grab, B. & Dixon, H. The transmission potential of monkeypox virus in human populations. *Int J Epidemiol* **17**, 643-650 (1988). https://doi.org/10.1093/ije/17.3.643
3. Jezek, Z. *et al.* Human monkeypox: a study of 2,510 contacts of 214 patients. *J Infect Dis* **154**, 551-555 (1986). https://doi.org/10.1093/infdis/154.4.551
4. Jezek, Z., Grab, B., Szczeniowski, M. V., Paluku, K. M. & Mutombo, M. Human monkeypox: secondary attack rates. *Bull World Health Organ* **66**, 465-470 (1988).
5. Rimoin, A. W. *et al.* Major increase in human monkeypox incidence 30 years after smallpox vaccination campaigns cease in the Democratic Republic of Congo. *Proc Natl Acad Sci U S A* **107**, 16262-16267 (2010). https://doi.org/10.1073/pnas.1005769107
6. Nolen, L. D. *et al.* Introduction of Monkeypox into a Community and Household: Risk Factors and Zoonotic Reservoirs in the Democratic Republic of the Congo. *Am J Trop Med Hyg* **93**, 410-415 (2015). https://doi.org/10.4269/ajtmh.15-0168
7. Whitehouse, E. R. *et al.* Clinical and Epidemiological Findings from Enhanced Monkeypox Surveillance in Tshuapa Province, Democratic Republic of the Congo During 2011-2015. *J Infect Dis* **223**, 1870-1878 (2021). https://doi.org/10.1093/infdis/jiab133
8. Wolff Sagy, Y. *et al.* Real-world effectiveness of a single dose of mpox vaccine in males. *Nat Med* **29**, 748-752 (2023). https://doi.org/10.1038/s41591-023-02229-3
9. Payne, A. B. *et al.* Incidence of Monkeypox Among Unvaccinated Persons Compared with Persons Receiving ≥1 JYNNEOS Vaccine Dose — 32 U.S. Jurisdictions, July 31–September 3, 2022. *MMWR. Morbidity and Mortality Weekly Report* **71**, 1278-1282 (2022). https://doi.org/10.15585/mmwr.mm7140e3
10. Payne, A. B. *et al.* Reduced Risk for Mpox After Receipt of 1 or 2 Doses of JYNNEOS Vaccine Compared with Risk Among Unvaccinated Persons-43 US Jurisdictions, July 31-October 1, 2022. *Mmwr-Morbidity and Mortality Weekly Report* **71**, 1560-1564 (2022).
11. Bertran, M. *et al.* Effectiveness of one dose of MVA-BN smallpox vaccine against mpox in England using the case-coverage method: an observational study. *Lancet Infect Dis* **23**, 828-835 (2023). https://doi.org/10.1016/S1473-3099(23)00057-9
12. Dalton, A. F. *et al.* Estimated Effectiveness of JYNNEOS Vaccine in Preventing Mpox: A Multijurisdictional Case-Control Study — United States, August 19, 2022–March 31, 2023. *MMWR. Morbidity and Mortality Weekly Report* **72**, 553-558 (2023). https://doi.org/10.15585/mmwr.mm7220a3
13. Deputy, N. P. *et al.* Vaccine Effectiveness of JYNNEOS against Mpox Disease in the United States. *N Engl J Med* **388**, 2434-2443 (2023). https://doi.org/10.1056/NEJMoa2215201
14. Rosenberg, E. S. *et al.* Effectiveness of JYNNEOS Vaccine Against Diagnosed Mpox Infection — New York, 2022. *MMWR. Morbidity and Mortality Weekly Report* **72**, 559-563 (2023). https://doi.org/10.15585/mmwr.mm7220a4
15. von Krempelhuber, A. *et al.* A randomized, double-blind, dose-finding Phase II study to evaluate immunogenicity and safety of the third generation smallpox vaccine candidate IMVAMUNE. *Vaccine* **28**, 1209-1216 (2010). https://doi.org/10.1016/j.vaccine.2009.11.030
16. Zitzmann-Roth, E. M. *et al.* Cardiac safety of Modified Vaccinia Ankara for vaccination against smallpox in a young, healthy study population. *PLoS One* **10**, e0122653 (2015). https://doi.org/10.1371/journal.pone.0122653
17. Pittman, P. R. *et al.* Phase 3 Efficacy Trial of Modified Vaccinia Ankara as a Vaccine against Smallpox. *N Engl J Med* **381**, 1897-1908 (2019). https://doi.org/10.1056/NEJMoa1817307
18. Greenberg, R. N. *et al.* A Multicenter, Open-Label, Controlled Phase II Study to Evaluate Safety and Immunogenicity of MVA Smallpox Vaccine (IMVAMUNE) in 18-40 Year Old Subjects with Diagnosed Atopic Dermatitis. *PLoS One* **10**, e0138348 (2015). https://doi.org/10.1371/journal.pone.0138348

19 Frey, S. E. *et al.* Safety and immunogenicity of IMVAMUNE(R) smallpox vaccine using different strategies for a post event scenario. *Vaccine* **31**, 3025-3033 (2013). https://doi.org/10.1016/j.vaccine.2013.04.050

20 Overton, E. T. *et al.* Safety and Immunogenicity of Modified Vaccinia Ankara-Bavarian Nordic Smallpox Vaccine in Vaccinia-Naive and Experienced Human Immunodeficiency Virus-Infected Individuals: An Open-Label, Controlled Clinical Phase II Trial. *Open Forum Infect Dis* **2**, ofv040 (2015). https://doi.org/10.1093/ofid/ofv040

21 Overton, E. T. *et al.* Immunogenicity and safety of three consecutive production lots of the non replicating smallpox vaccine MVA: A randomised, double blind, placebo controlled phase III trial. *PLoS One* **13**, e0195897 (2018). https://doi.org/10.1371/journal.pone.0195897

22 Ilchmann, H. *et al.* One- and Two-Dose Vaccinations With Modified Vaccinia Ankara-Bavarian Nordic Induce Durable B-Cell Memory Responses Comparable to Replicating Smallpox Vaccines. *J Infect Dis* **227**, 1203-1213 (2023). https://doi.org/10.1093/infdis/jiac455

23 Greenberg, R. N. *et al.* A Randomized, Double-Blind, Placebo-Controlled Phase II Trial Investigating the Safety and Immunogenicity of Modified Vaccinia Ankara Smallpox Vaccine (MVA-BN(R)) in 56-80-Year-Old Subjects. *PLoS One* **11**, e0157335 (2016). https://doi.org/10.1371/journal.pone.0157335

24 Bavarian Nordic. *A Phase II Trial to Compare a Liquid-frozen and a Freeze-dried Formulation of IMVAMUNE (MVA-BN®) Smallpox Vaccine in Vaccinia-naïve Healthy Subjects*, <https://clinicaltrials.gov/study/NCT01668537> (2020).

25 Frey, S. E. *et al.* Comparison of lyophilized versus liquid modified vaccinia Ankara (MVA) formulations and subcutaneous versus intradermal routes of administration in healthy vaccinia-naive subjects. *Vaccine* **33**, 5225-5234 (2015). https://doi.org/10.1016/j.vaccine.2015.06.075

26 Overton, E. *et al.* A randomized phase 3 trial to assess the immunogenicity and safety of 3 consecutively produced lots of freeze-dried MVA-BN(R) vaccine in healthy adults. *Vaccine* **41**, 397-406 (2023). https://doi.org/10.1016/j.vaccine.2022.10.056

27 Jackson, L. A. *et al.* Safety and immunogenicity of a modified vaccinia Ankara vaccine using three immunization schedules and two modes of delivery: A randomized clinical non-inferiority trial. *Vaccine* **35**, 1675-1682 (2017). https://doi.org/10.1016/j.vaccine.2017.02.032

28 Bunge, E. M. *et al.* The changing epidemiology of human monkeypox-A potential threat? A systematic review. *PLoS Negl Trop Dis* **16**, e0010141 (2022). https://doi.org/10.1371/journal.pntd.0010141

29 Gelman, A. *et al.* Bayesian Data Analysis. (2013). https://doi.org/10.1201/b16018
