# Supplementary material for: Predicting vaccine effectiveness for mpox
Source: Nat Commun. 2024 May 8;15:3856. doi: 10.1038/s41467-024-48180-w (PMC11078999; doi:10.1038/s41467-024-48180-w)
Supplement: Supplementary file 3 — Reporting Summary [file 41467_2024_48180_MOESM3_ESM.pdf]

nature portfolio

Corresponding author(s): David Khoury

Last updated by author(s): Apr 5, 2024

Reporting Summary

Nature Portfolio wishes to improve the reproducibility of the work that we publish. This form provides structure for consistency and transparency in reporting. For further information on Nature Portfolio policies, see our Editorial Policies and the Editorial Policy Checklist.

Statistics

For all statistical analyses, confirm that the following items are present in the figure legend, table legend, main text, or Methods section.

n/a	Confirmed	
☐	☒	The exact sample size (*n*) for each experimental group/condition, given as a discrete number and unit of measurement
☒	☐	A statement on whether measurements were taken from distinct samples or whether the same sample was measured repeatedly
☐	☒	The statistical test(s) used AND whether they are one- or two-sided<br>*Only common tests should be described solely by name; describe more complex techniques in the Methods section.*
☐	☒	A description of all covariates tested
☐	☒	A description of any assumptions or corrections, such as tests of normality and adjustment for multiple comparisons
☐	☒	A full description of the statistical parameters including central tendency (e.g. means) or other basic estimates (e.g. regression coefficient) AND variation (e.g. standard deviation) or associated estimates of uncertainty (e.g. confidence intervals)
☐	☒	For null hypothesis testing, the test statistic (e.g. *F*, *t*, *r*) with confidence intervals, effect sizes, degrees of freedom and *P* value noted<br>*Give P values as exact values whenever suitable.*
☐	☒	For Bayesian analysis, information on the choice of priors and Markov chain Monte Carlo settings
☐	☒	For hierarchical and complex designs, identification of the appropriate level for tests and full reporting of outcomes
☒	☐	Estimates of effect sizes (e.g. Cohen's *d*, Pearson's *r*), indicating how they were calculated

Our web collection on statistics for biologists contains articles on many of the points above.

Software and code

Policy information about availability of computer code

Data collection	For data on vaccine effectiveness, we extracted the case/control incidence (number of events) data reported in each study disaggregated by timepoint, age or region where possible. For immunogenicity data, we contacted the sponsor by e-mail to request access to the de-identified individual-level data presented in the published work. This request was denied (27 March 2023). Therefore, we extracted summary data (Geometric Mean Titer (GMT) and confidence intervals (CI)) from tables (where available) or figures using WebPlotDigitiser (https://automeris.io/WebPlotDigitizer, version 4.6). Where data was extracted from an image, two individuals (MB, SRK) extracted the data independently with the geometric mean of the two extracted values used and we confirmed that discrepancies between extracted values were always less than 1%.
Data analysis	All meta-analyses and meta-regression were performed using a hierarchical Bayesian data analysis framework in RStan using the default HMC sampler (RStan: The R interface to Stan v. 2.26.23 (2023). https://mc-stan.org/). Code is available here: https://github.com/iap-sydney/Mpox_ELISA_Effectiveness_correlates

For manuscripts utilizing custom algorithms or software that are central to the research but not yet described in published literature, software must be made available to editors and reviewers. We strongly encourage code deposition in a community repository (e.g. GitHub). See the Nature Portfolio guidelines for submitting code & software for further information.

Data

Policy information about availability of data

All manuscripts must include a data availability statement. This statement should provide the following information, where applicable:

- Accession codes, unique identifiers, or web links for publicly available datasets
- A description of any restrictions on data availability
- For clinical datasets or third party data, please ensure that the statement adheres to our policy

All extracted data are available in the GitHub repository: https://github.com/iap-sydney/Mpox_ELISA_Effectiveness_correlates

Research involving human participants, their data, or biological material

Policy information about studies with human participants or human data. See also policy information about sex, gender (identity/presentation), and sexual orientation and race, ethnicity and racism.

Reporting on sex and gender	NA - meta-analysis - only aggregate data from each study was available
Reporting on race, ethnicity, or other socially relevant groupings	NA - meta-analysis - only aggregate data from each study was available
Population characteristics	Population characteristics, regarding study location, vaccines used are reported in Table S1.
Recruitment	NA - meta-analysis - no participants were directly recruited in this study.
Ethics oversight	This work was approved under the UNSW Sydney Human Research Ethics Committee (approval HC200242).

Note that full information on the approval of the study protocol must also be provided in the manuscript.

Field-specific reporting

Please select the one below that is the best fit for your research. If you are not sure, read the appropriate sections before making your selection.

- [x] Life sciences
- [ ] Behavioural & social sciences
- [ ] Ecological, evolutionary & environmental sciences

For a reference copy of the document with all sections, see nature.com/documents/nr-reporting-summary-flat.pdf

Life sciences study design

All studies must disclose on these points even when the disclosure is negative.

Sample size

The sample sizes (number participants) for all effectiveness and immunogenicity studies are provided in Table S1 and S2. The primary studies Since the studies aggregated in this meta-analysis were observational studies of vaccine effectiveness - sample size of the studies was dependent on the populations being followed in secondary contact studies or identified in health data-bases. For the immunogenicity studies , where sample size justification is provided it is detailed in the clinical trial registration at the following codes (clinicaltrials.gov):

NCT00189956
NCT00316524
NCT01913353
NCT00316602
NCT00437021
NCT00316589
NCT01144637
NCT00686582
NCT00857493
NCT01668537
NCT00914732
NCT03699124
NCT01827371

Data exclusions	Studies were excluded if they did not meet inclusion criteria of systematic search (detailed in supplementary material). Of included studies all data was extracted and included in analysis.
Replication	Data extracted from figures was verified by two authors, and values were highly concordant.
Randomization	This study performs a meta-analysis of all available studies of Vaccinia-based vaccine effectiveness against mpox, no RCTs were identified, only non-RCT observational studies.

Blinding	NA - meta-analysis of observational (non-RCT) clinical trials.

Reporting for specific materials, systems and methods

We require information from authors about some types of materials, experimental systems and methods used in many studies. Here, indicate whether each material, system or method listed is relevant to your study. If you are not sure if a list item applies to your research, read the appropriate section before selecting a response.

Materials & experimental systems

n/a	Involved in the study
☒	☐ Antibodies
☒	☐ Eukaryotic cell lines
☒	☐ Palaeontology and archaeology
☒	☐ Animals and other organisms
☒	☐ Clinical data
☒	☐ Dual use research of concern
☒	☐ Plants

Methods

n/a	Involved in the study
☒	☐ ChIP-seq
☒	☐ Flow cytometry
☒	☐ MRI-based neuroimaging

Plants

Seed stocks	NA
Novel plant genotypes	NA
Authentication	NA
